# Technique for retrospective respiratory and cardiac-gated phase contrast flow measurements

**DOI:** 10.1186/1532-429X-14-S1-W26

**Published:** 2012-02-01

**Authors:** Ashley G Anderson, Eric M Schrauben, Kevin Johnson, Oliver Wieben

**Affiliations:** 1 Medical Physics, University of Wisconsin - Madison, Madison, WI, USA; 2 Radiology, University of Wisconsin - Madison, Madison, WI, USA

## Summary

A technique for evaluation of respiratory impact on cardiac-gated phase contrast flow acquisitions is proposed. An example study was performed showing the effect of active respiration on CSF flow through the spinal canal.

## Background

The effect of the respiratory cycle on flow is well documented for cerebrospinal venous return [1] and CSF flow [2]. However, cranial phase contrast (PC) MR flow measurements neglect the influence of respiration. Breath-held acquisitions are possible, but mask physiological flow changes that occur during active respiration. Here we demonstrate a technique to evaluate the effects of active respiration on CSF flow measurements throughout the cardiac cycle for 2 respiration states.

## Methods

**Figure 1:**
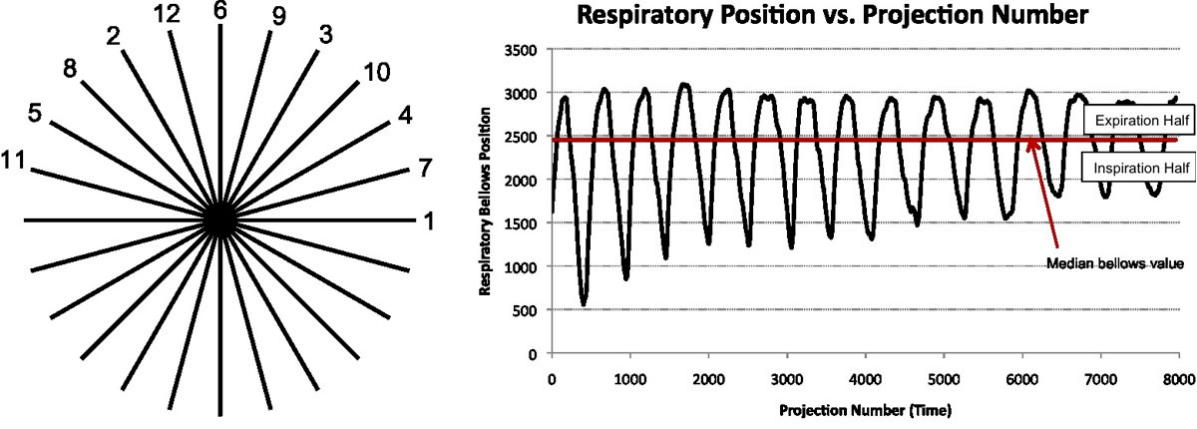
(Left) Acquisition order for a single, 12-projection 2D radial interleaf. Acquisition order in each interleaf is determined by a bit-reversal pattern that provides samples l-space in a relatively evenly distributed pattern. Each interleaf consists of as many projections as are predicted to fit in a heartbeat. (Right) Respiratory position as a function of projection number. In this case projection number corresponds to the actual acquisition order of the projections. Thus this may be seen as a plot of respiratory position vs. time. The period of the respiratory cycle is significantly slower than that of the cardiac cycle, so interference with the acquisition order is not an issue. Respiratory position here is based on a bellows system, but could also come from a navigator signal, center-of-mass analysis, or other respiratory motion estimation method.

Our approach relies on pseudo-random sampling of radial projections, which allows reconstruction of subsets with little artifact due to even filling of k-space (Fig. 1a). Cardiac triggers and respiratory position are recorded throughout the acquisition. Each projection then has a corresponding cardiac position (time since last trigger) and respiratory position. In the current implementation, all projections are sorted into 2 respiratory phases based on the median respiratory position. Each phase corresponds to the plateau surrounding end-inspiration or -expiration. Each respiratory phase is represented by a cardiac gated image series that is reconstructed using a temporal filtering similar to view sharing in Cartesian acquisitions [3].

Three volunteers were scanned on a clinical 3T system with 2D radial PC acquisitions [4] between the C2 and C3 vertebrae: TR/TE = 9.4/6.1 ms, tip = 5°, resolution = 0.9x0.9x5 mm, and Venc = 8cm/s. Data were acquired during inspiration and expiration breath holds (1500 projections, 30 s), and during regular free and deep breathing (8000 projections, 2:32 min). Triggering was accomplished with pulse oximeter and bellow signals.

## Results

**Figure 2:**
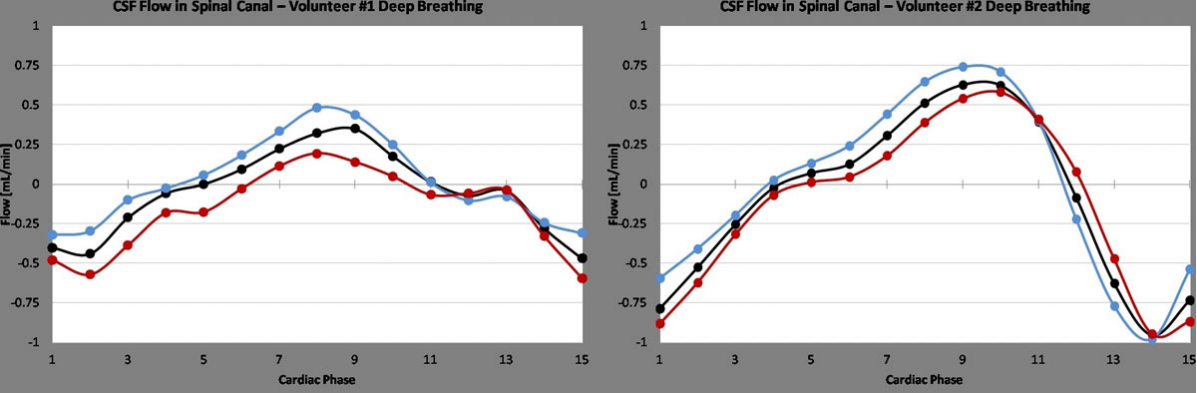
– CSF flow through the spinal cord for two volunteers during deep breathing. Three curves are shown for each volunteer, all measured from the same acquisition. Each chart depicts the flow from the respiratory phase surrounding inspiration (red) and expiration (blue) as well as the average flow measured without respiratory binning (black). Note the increase in flow in the inspiration phase, which corresponds to a decrease in thoracic pressure.

Fig. 2 shows the resulting flow waveforms acquired during deep breathing for two volunteers. Though the waveforms show variation, the trend of increased flow during inspiration holds in both examples. Average forward (Qf), reverse (Qr), and net (Qnet) flow were calculated for both phases in the free breathing scans. For the inspiration phase, these values were: Qf = 3.06, Qr = 1.53, and Qnet = 1.53 ml/min. For the bin surrounding end-expiration, these values were: Qf = 2.53, Qr = 2.02, Qnet = 0.51 ml/min.

## Conclusions

This study shows the feasibility of double retrospectively gated PC MR imaging for assessing flow in respiratory phases. The reconstruction scheme can be easily adjusted to capture additional respiratory phases at the expense of additional scan time. Our initial results demonstrate changes in CSF flow due to respirator phase, namely an overall increase in forward flow, as well as a decrease in reverse flow, leading to a significant increase in net flow during inspiration. This method can also be used to assessing venous and other flow affected by respiration.

## Funding

NIH NHLBI R01HL072260.
